# Time to rechallenge primary prevention ICD guidelines

**DOI:** 10.1002/ehf2.14113

**Published:** 2022-08-24

**Authors:** Bethany Wong, Kenneth McDonald, David Keane

**Affiliations:** ^1^ UCD School of Medicine Health Sciences Centre Belfield Dublin Ireland; ^2^ Cardiology Department St Vincent's Hospital Elm Park Dublin Ireland

## Modern optimal medical therapy

Patients, therapy, and evidence have evolved in leaps since 2002, when MADIT‐2^1^ first demonstrated a mortality benefit from using primary prevention ICD therapy. From 1995 to 2014, there has been a 44% decline in sudden death across trials.^2^ This is attributed to an improvement in adherence to heart failure (HF) medical therapy, namely, angiotensin inhibition (ACEI/ARB), beta‐blockers, and mineralocorticoid receptor antagonists (MRA).^2^ There is also a contemporary shift in the demographics of HF patients, who are older and have more co‐morbidities, leading to a competing risk of non‐sudden cardiac death (SCD). In addition to medical therapy and lifestyle modification, structural improvements in hospital and community pathways to access early HF specialist input have also advanced.^3^

In addition to established therapies, landmark trials using sacubitril–valsartan^4^ (ARNi) and sodium–glucose co‐transporter 2 inhibitors^5^ (SGLT2i) have each demonstrated a further ~20% reduction in ventricular arrhythmia (VA) and SCD. There is now evidence to support that ARNi use improves cardiomyocyte electrophysiological remodelling with a reduction in QTc, QRS duration, and mechanical dispersion at 6 months.^6^ SGLT2i also act on multiple electrophysiological characteristics (viz. Ca^2+^ regulation, late Na^+^ and Na^+^/hydrogen‐exchanger currents),^7^ which may similarly contribute to their anti‐arrhythmic properties outside of their ability to improve left ventricular ejection fraction (LVEF). Recent observational evidence demonstrates that patients who receive guideline‐directed medical therapy have an almost fourfold reduction in risk of death at 2 years compared with those not on therapy, conferring a 34% reduction in risk of death for each drug added in patients who have an ICD implanted.^8^

Current ESC guidelines^9^ recommend ICD implantation with a Class 1a indication, in those with ischaemic HF with an LVEF <35% despite optimal medical therapy (OMT) for ≥3 months, symptoms (NYHA II/III), a QRS < 130 ms, and a life expectancy of at least 1 year. Considering there have been no new ICD trials in ischaemic HF since SCD‐HeFT,^10^ published in 2005, these recommendations seem outdated. For non‐ischaemic HF, new evidence from the DANISH trial softened the ICD indication from a Class I indication in 2016 guidelines to a IIa classification in 2021.

The recommendation of OMT for ≥3 months before ICD implantation may be premature. Post hoc analyses of ARNi therapy suggest that there are still improvements in LVEF well beyond 3 months. At 6 months after ARNi initiation, 32% of ICD eligible patients at baseline become ineligible due to LVEF improvements, and by 12 months, that proportion almost doubles to 62%.^4^ ICD implantation at 3 months would, in majority of patients, be too soon, as their LVEF would still be on the upward trajectory, with many being able to avoid an ICD if given more time for left ventricular recovery. This is likely also the case for improvements in NYHA functional classification. ESC guidelines advise that those with NYHA Class I do not benefit from primary prevention ICD therapy. ARNi therapy improves NYHA class by a mean difference of −0.79^11^ and SGLT‐2i by an odds ratio of 1.3,^12^ and ~70% of patients enrolled in PARADIGM and EMPEROR‐reduced were categorized as NYHA Class II. This suggests a proportion of NYHA Class II patients, treated with ARNi and SGLT‐2i therapy improve to below NYHA Class II, thereby no longer meeting ICD indication under the current guidelines. These benefits have been recently demonstrated in a multi‐centre Italian registry,^13^ and furthermore, the benefit of SGLT2i on VA/SCD risk have been demonstrated to be incremental past 12 months.^5^

Delaying implantation and thereby reducing the number of unnecessary ICD implants would not only reduce patient risk associated with implantation (5–10% risk of infection/ pneumothorax/ lead displacement^14^) and inappropriate shocks (~20% lifetime risk^15^) but also improve patient psychological stress caused by fear of inappropriate shocks. Additionally, reducing implantation of unnecessary ICDs would be cost‐saving, not only for the device cost, generator replacements and initial complications (de novo device infection costs ~€23 000^16^) but also for follow‐up pacing clinics as well as patient travel and convenience.

The counter argument in favour of early implantation is that some patients while awaiting for NYHA/LVEF improvements beyond 3 months may have a fatal arrhythmia, but by implementing effective personalized risk stratification, this would be minimised.

## Risk stratification

Prolongation of QRS is associated with increased SCD risk.^17^ Guidelines recommend those with a QRS > 150 ms with LBBB to be treated with CRT‐D therapy, with strong evidence of morbidity and mortality benefit.^9^ For those patients with a narrow QRS, there is less evidence of the benefit of a defibrillation device. MADIT‐2 demonstrated that despite the majority of enrolled patients having a QRS duration of <150 ms, the mortality reduction in these patients treated with ICD was not statistically significant.^1^ This therefore leads to the question: Would all patients with HFREF, a narrow QRS complex and on current OMT, benefit from an ICD? Or perhaps, only subgroups who are high risk should be offered ICD therapy?

There is emerging evidence of patient characteristics, imaging and biomarkers that may aid future stratification of those most likely to benefit from primary prevention ICD therapy. The DANISH trial, published in 2016, found no all‐cause mortality benefit to ICD therapy in those with non‐ischaemic cardiomyopathy. This trial was before the widespread use of ARNi and SGLT2i. In post hoc propensity‐matched analyses of PARADIGM‐HF,^18^ ARNi in addition to ICD use was found to have a larger impact on SCD in non‐ischaemic cardiomyopathy compared with those with an ischaemic aetiology. This reinforces the improved risk of those with a non‐ischaemic aetiology who are on current OMT.

Cardiovascular magnetic resonance (CMR) has emerged as an important tool for VA risk assessment. Presence and burden of myocardial fibrosis (a well‐established substrate for VA) using late gadolinium enhancement has become more widely accessible and utilized.^19^ CMR GUIDE^20^ is an important ongoing randomized controlled trial, which plans to use CMR to identify fibrosis and randomize patients with mild to moderately impaired LV systolic function (LVEF 36–50%) to ICD vs. implantable loop recorder (ILR). The primary endpoint will be SCD/VA. This study may further highlight the importance of myocardial fibrosis as a risk factor, independent to LVEF.

Elevated NT‐proBNP has also been shown to increase the likelihood of VA/SCD and therefore is an important stratification marker for those most likely to benefit from ICD therapy. This has also been shown in DAPA‐HF post hoc analyses,^5^ demonstrating that NT‐proBNP was the largest predictor of VA/SCD outside of previously documented VA (i.e. already had an indication for secondary prevention ICD therapy). As panels of biomarkers become cheaper and more accessible, additional biomarkers such as ST2 and galectin‐3 may become standard practice for composite biomarker risk stratification.

Although the EU‐CERT‐ICD^21^ controlled multicentre cohort study recently showed a mortality benefit to ICD therapy, the patient cohort was not on contemporary OMT. It did however highlight two important non‐benefitting subgroups: those with diabetes and those aged >75 years old. Therefore, as the HF population ages with more co‐morbidities such as diabetes, this may reduce the overall benefit of primary prevention ICD.

ILRs provide the advantage of continuous monitoring of cardiac rhythm over a 3‐year period with daily remote transmission. It is acknowledged that they currently do not deliver direct therapy in the event of a VA, resulting in SCD; however, they enable early detection for secondary VA prevention, and in the future, they may be able to immediately notify the nearest emergency service in the case of sustained VA. The future of non‐invasive monitoring devices, which includes photoplethysmography technology in wrists‐worn devices,^22^ is showing promise, at their ability to monitor for life‐threatening arrhythmias. As these become more widely validated and available, these are likely going to be central to appropriate risk stratification.

SCD risk models such as the Seattle Proportional Risk Model have been validated in large cohorts.^23^ Despite its limitations (developed in 2015, before the widespread use of sacubitril–valsartan/SGLT2‐i and does not incorporate CMR), it identifies patient characteristics such as a younger age, being male and those with an elevated BMI that confer a higher SCD risk and, conversely, those with diabetes and renal dysfunction who are at lower risk. This risk model could be incorporated into ICD therapy decisions, rather than using solely LVEF/NYHA, but are also not included in current 2021 ESC guidelines. We therefore feel these guidelines offer limited insight into personalisation of risk/benefit of ICD therapy in the current era of modern HF management.

## Future considerations

Although our perspective may not be welcomed by ICD manufacturers or some implanting physicians, it is time for a trial to determine whether today, primary prevention ICD implantation would still convey any prognostic benefit in ischaemic and non‐ischaemic HFREF, in patients with a narrow QRS duration in the current era of disease‐modifying therapy. Such a trial could also identify a more refined risk stratification system (rather than LVEF and NYHA classification alone) such as distribution of fibrosis on CMR (size, location, and extent), use of wearable technology, patient characteristics, and biomarkers. Such a trial could also offer guidance on timing of ICD therapy. It could be conceived that close arrhythmia monitoring with ILR or future wearable technology could be the most effective strategy in high‐risk patients on modern OMT. There are no trials planned in intermediate/high‐risk HFREF patients; however, the planned PROFID project,^24^ which will compare low risk HFREF patients to OMT, with and without ICD, is currently recruiting with results hopefully in 2025. Additionally, this EU‐funded randomized open‐label trial will also challenge the use of LVEF and risk of VA/SCD, by a second study, randomizing those with an LVEF >35% who are at high risk of SCD, to an ICD alongside OMT. This trial, we hope will provide more robust evidence towards a more personalized and effective approach to primary prevention ICD therapy. Until then, we are left with an unsettling feeling of whether old evidence still holds true for implanting primary prevention ICDs in an older HF population in the current era of OMT.

## Conflict of interest

K McDonald: Consulting fees for Novartis.

## Funding

This work was performed within the Irish Clinical Academic Training (ICAT) Programme, supported by the Wellcome Trust and the Health Research Board (Grant Number 203930/B/16/Z), the Health Service Executive, National Doctors Training and Planning, and the Health and Social Care, Research and Development Division, Northern Ireland.
